# When Breathing Becomes a Challenge: A Case of Congenital Myasthenia Gravis in an Indian Neonate With a DOK-7 Gene Mutation

**DOI:** 10.7759/cureus.38842

**Published:** 2023-05-10

**Authors:** Vaidehi Mendpara, Sanjay Bethanabotla, Megha Yadav, Vaishnavi Kanisetti, Gurpreet Singh, Abhirami Das, Sweta Sahu, Hitesh Patel

**Affiliations:** 1 Medicine and Surgery, Pediatrics, Government Medical College Surat, Surat, IND; 2 Internal Medicine, Osmania Medical College, Hyderabad, IND; 3 Medicine and Surgery, Maharani Laxmi Bai Medical College, Jhansi, IND; 4 Medicine and Surgery, Bhaskar Medical College, Hyderabad, IND; 5 Medicine, Government Medical College and Hospital, Amritsar, IND; 6 Internal Medicine, Rajiv Gandhi Medical College, Thane, IND; 7 Surgery, Jagadguru Jayadeva Murugarajendra (JJM) Medical College, Davanagere, IND; 8 Pediatrics and Child Health, Government Medical College and Hospital, Surat, IND

**Keywords:** respiratory muscle weakness, neonatal ventilation, salbutamol, respiratory distress, neuromuscular junction disorder, dok-7 mutation, congenital myasthenia gravis

## Abstract

A rare neuromuscular condition known as congenital myasthenia gravis (CMG) affects some people from birth or very soon after. It results in fatigue and muscle weakness because of genetic abnormalities that interfere with the neuromuscular junction's ability to function, where the nerves and muscles connect. Even among those who have the same genetic mutation, the severity of CMG symptoms might differ considerably. The most typical signs of CMG include eyelid drooping, breathing issues, muscle weakness and weariness, and difficulties swallowing. Clinical examinations, neurophysiologic tests, and genetic analyses are frequently combined to make the diagnosis of CMG. Although there is no known treatment for CMG, many patients may control their symptoms and lead relatively normal lives with the right care.

A newborn with CMG due to a DOK-7 gene mutation is described in this article, along with its very early onset. The DOK-7 mutation is a rare variant in the Indian population that causes CMG and usually manifests as 'limb girdle' weakness. However, due to muscle weakness, the neonate in this case developed severe respiratory distress and later died despite rigorous life-saving measures.

## Introduction

Myasthenia gravis is an autoimmune disease that typically presents in the third to fifth decade of life in women and the sixth to eighth decade of life in men with abnormal levels of fatigue on exertion. Paediatric myasthenia, or myasthenia affecting children under the age of 18, accounts for only about 10% of the global myasthenia burden [1]. Congenital myasthenia gravis can typically present in three main forms: transient neonatal myasthenia, where maternal antibodies directed against the acetylcholine receptor (AChR) or less often anti-muscle specific kinase (MuSK) antibodies cross over into the foetal circulation transplacentally and cause symptoms of weakness and poor feeding right after birth that can resolve spontaneously within weeks to two months of being born [2]. Congenital myasthenia syndrome (CMS) is the second mode of presentation and comprises an array of inherited disorders characterized by genetic defects that affect proteins present at the neuromuscular junction [3]. Juvenile myasthenia gravis is the third type of presentation, which is similar to adult-onset myasthenia in the clinical features and pathophysiology but presents at a much earlier age. Congenital myasthenia syndromes have been classified in many different ways based on the inheritance as autosomal recessive, which forms the majority, or autosomal dominant; as pre-synaptic, synaptic, and post-synaptic CMS; based on the function of the mutated proteins; glycosylation defects; and also based on the long term prognosis [4]. However, the overarching clinical features remain similar and are characterized by varying degrees of fatigable weakness presenting soon after birth affecting the limb, ocular, and bulbar muscles, resulting in hypotonia, ptosis, and difficulty feeding. More seriously, it can also present with periods of apnea, cyanosis, and respiratory distress precipitated by triggers such as infections [5]. CMS is characterized by the absence of anti-AChR and anti-MuSK antibodies in the serum [6]. The prognosis of CMS varies based on the genetic defect, and the treatment includes agents like pyridostigmine, a cholinergic agonist that inhibits acetylcholinesterase, adrenergic agonists such as ephedrine and albuterol, and open channel blockers of AChR such as fluoxetine, which are useful in certain subtypes of CMS [7]. In this case, we gave a trial of salbutamol, which is a beta agonist, to help relieve respiratory symptoms in the patient.

So far, 12 genes have been identified that code for proteins involved in neuromuscular transmission. Despite extensive testing, approximately half of the CMSs have yet to be identified molecularly. DOK7 mutations were first identified in recessive forms of CMS in 2006 [8]. In this case, we have diagnosed a neonate with atypical presentations of respiratory symptoms and a DOK-7 mutation in India.

## Case presentation

A full-term baby was delivered to a G4P1A2L1 mother by vacuum-assisted vaginal delivery and who cried shortly after birth with APGAR scores of 8 and 9 at one and five minutes, respectively. The baby did not require any additional help breathing and was roomed in with the mother. The mother felt normal fetal movements during her antenatal period, and it was a well-followed pregnancy with normal antenatal sonography. Within three to four hours, the baby developed a bluish discoloration of the skin and had difficulty breastfeeding, which prompted the mother to seek medical attention from a pediatrician. A physical examination revealed that the infant had central cyanosis (oxygen saturation [SpO2] of 70-83% on room air), and the child was transferred to the neonatal intensive care unit (NICU) for further treatment.

**Figure 1:**
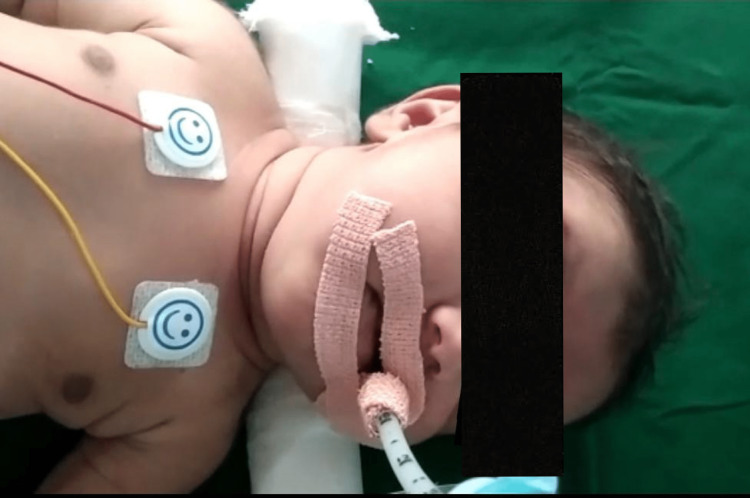
The neonate was kept on SIMV support. Due to worsening respiratory distress, the neonate was given synchronized intermittent mandatory ventilation (SIMV) to maintain oxygen saturation.

In the NICU, the baby was hypotonic, cyanosed, and had a capillary refill test of less than three seconds. The temperature was normal, and the pulses were felt very clearly in all four limbs. Respiratory findings were consistent with rhythmic and jerky breathing with intermittent subcostal retraction, air entry bilaterally equal, and a respiratory rate of 20-24 breaths per minute. A cardiac examination revealed a normal heart sound without any abnormal cardiac murmurs. Neurological status showed a hypotonic baby. The patient responded to tactile stimulation but was lethargic and drowsy. There was an absence of any active limb movement (Video 1, Video 2). The abdominal examination was normal and negative for any organomegaly. SpO2 was around 80-85% on room air, which rose to 90-100% when provided with 100% oxygen via a hood box. However, frequent desaturation occurred during episodes of apnea, so the baby was given continuous positive airway pressure (CPAP) support (Video 3). After about an hour, the baby was kept on synchronized intermittent mandatory ventilation (SIMV) ventilation due to its deteriorating condition (Figure 1).

**Video 1:** Neonate on admission The neonate was lethargic and had a weak cry with an absence of any active limb movement

**Video 2:** Neonate with visible signs of respiratory distress Respiratory findings were consistent with rhythmic and jerky breathing with intermittent subcostal retraction.

**Video 3:** Neonate on CPAP The baby was given continuous positive airway pressure (CPAP) support to maintain oxygen saturation.

According to the additional tests that were performed, the complete blood count, C-reactive protein (CRP), and renal function tests (RFT) were all within normal limits. The blood gas reports indicated that respiratory acidosis was present (Table 1), and the 2D ECHO, EEG, and USG brain were all normal. Blood tests to rule out inborn metabolic errors were done along with genomic sequencing. After consulting with a neurophysician, syrup piracetam was started, considering it to be a breath-holding spell. Muscular pathology was suspected due to symptoms such as difficulty feeding, a weak cry, respiratory distress, and muscle weakness. Following additional examinations, genetic testing was agreed upon to rule out myasthenic pathology. A genetic test confirmed the suspicion of CMS due to the DOK-7 mutation (Table 2). According to one study, oral salbutamol is well tolerated and improves muscle function in children with DOK7 CMS. Therefore, a trial of oral salbutamol (2-4 mg three times a day) was initiated.

**Table 1:** Blood gas analysis monitored after neonate's admission The blood gas analysis showed acidosis picture due to worsening respiratory distress. PO2: partial pressure of oxygen, PCo2: partial pressure of carbon dioxide, Hco3: bicarbonate, SPO2: oxygen saturation

Day	1st	2nd	3rd	4th	5th	6th	7th	8th	9th	10th	11th
PH	7.13	7.4	7.6	7.3	7.4	7.3	7.5	7.4	7.2	7.1	7.3
PO2(mmHg)	40	146	242	89	79	42	44	59	61	23	68
PCo2(mmHg)	102.4	26.5	19.2	40	32.4	35	21.2	31.5	74.3	86	108
Hco3(mEq/L)	33.4	24.2	21.7	21.7	19.6	19.7	19.1	19.7	24.2	30.7	47
SPO2(%)	54.5	99.6	99.9	96.5	95.8	76.2	87	91.1	84.5	25.7	89
BE[base excess](mmol/L)	3.6	1.9	3.1	-2.9	-4.4	-4.7	-2.9	-3.8	1.7	2.9	24.8

**Table 2:** Whole genome sequencing report showed heterozygous DOK-7 mutation. The genomic sequence report showed likely compound heterozygous variants causative of the reported phenotype.

Gene & Transcript	Variant	Location	Zygosity	Disorder(OMIM)	Inheritance
DOK7 NM_001301071.2	c.962C>T (p.Pro321Leu)	Exon 7	Heterozygous	Myesthenic Syndrome, Congenital, 10(254300)	Autosomal recessive
DOK7 NM_001301071.2	c.1793C>A (p.Ser598Ter)	Exon 10	Heterozygous	Myesthenic Syndrome, Congenital, 10(254300)	Autosomal recessive

The patient was started on an extubation trial and weaned off to an high-flow nasal cannula (HFNC). However, soon the patient had CO2 narcosis and respiratory acidosis. Despite ventilator support, the patient's condition deteriorated, and the baby died on the 12th day.

## Discussion

Congenital myasthenic syndromes are a group of rare, inherited neuromuscular disorders characterized by dysfunction of the neuromuscular junction. The onset age of CMS is highly variable, ranging from early childhood with apneic attacks and respiratory difficulties to adulthood with limb-girdle weakness and ptosis [8]. Children with CMS typically present with the condition during the first two years of life, and many of them exhibit symptoms as early as the neonatal period or early infancy. Patients may show symptoms such as hypotonia, facial weakness, swallowing difficulties, respiratory dysfunction, ptosis, and ophthalmoparesis. In this case, the presentation of symptoms was very immediate, i.e., three to four hours after birth, and respiratory symptoms were prominent. Joshi et al. [9] reported a case of a child with bilateral ptosis, a weak gag reflex, generalized hypotonia with weakness of the intercostal muscles and diaphragm, and depressed deep tendon reflexes, where the child presented with drooping of the eyelids at six months. Another case [10] was reported of a six-month-old patient who presented with episodic respiratory distress and apnea since the 10th day and was diagnosed with congenital myasthenia gravis based on electrodiagnostic findings.

CMS inheritance can be either autosomal dominant or recessive. CMS can have presynaptic, synaptic basal laminar, or postsynaptic neuromuscular defects [11]. Depending on the type of CMS, the deficient protein can be Dok-7, Musk, b2-laminin, AChR, GFPT1, or Plectin [12]. Around 85%-90% of known pathogenic variants in genes such as CHRNE (50%, including autosomal dominant and recessive variants), RAPSN (15%-20%), DOK7 (10%-15%), COLQ (10%-15%), and GFPT1 (2%) are found in India. As a result, DOK-7 is one of the rare variants of CMS in an Indian patient [13]. The DOK7 gene is responsible for encoding the Dok-7 protein on chromosome 4 p16.2 [14]. DOK7 mutations result in the loss of two phosphorylated tyrosine residues that recruit CRK proteins, which are essential for acetylcholine receptor anchoring at synapses [15].

The patient exhibited mutations in DOK7 (downstream of kinase), an adapter protein for MuSK, which is an important cause of congenital myasthenia. The presentation of CMS with DOK7 mutations can differ greatly, with most patients having a ‘limb girdle’ pattern of weakness (proximal muscles are more affected than distal muscles), and an altered waddling gait after achieving normal milestones. Ptosis may be present, but eye movements are rarely involved [16]. Fatigability may not be present or might be difficult to detect in neonates. Feeding difficulties, hypotonia, and stridor are diagnostic clues that indicate further neurological evaluation is required. In a survey of 79 CMS patients, the DOK7 mutation carriers had the worst outcomes [17]. Six of the eight ventilated, disabled individuals had the DOK7 variant [17]. Our patient presented with lethargy, hypotonia, and difficulty breathing within the first three to four hours of life. Due to the rarity of the disease and lack of sufficient research, treatment modalities for congenital myasthenia are often symptomatic [4]. Treatment with acetylcholinesterase inhibitors is futile and may often worsen symptoms. Ephedrine has been used in the treatment of CMS with DOK7 mutations. Due to the adverse alpha-adrenergic effects, beta-2 agonists such as salbutamol are being used as first-line treatments. Beta-2 agonists have been shown to improve symptoms in CMS associated with DOK7 mutations. In nine infants with DOK7 congenital myasthenic syndrome (CMS), oral salbutamol administered for at least one month improved muscle function without causing significant adverse effects, with all participants reporting an increase in stamina and function [18]. Beta-2 agonists increase cAMP kinase-A activity, which compensates for the reduced signaling of MuSK due to the DOK7 mutation. The improvement in muscle function seen with salbutamol is comparable to what has been reported with ephedrine, most likely owing to the beta-2 adrenergic effects [19]. However, since ephedrine has both alpha and beta adrenergic effects, there are concerns about its cardiac and central side effects with long-term use in children. Albuterol has also been shown to significantly and persistently enhance muscle strength in patients with DOK7 congenital myasthenic syndrome [20]. Our patient was given a trial of salbutamol (2-4 mg three times a day) in an attempt to improve muscle function.

Up until now, close to 90 cases with the DOK-7 mutation CMS have been reported in the literature (Table 3). Hence, this case becomes reportable as DOK-7 is a rare variant of a genetic mutation causing CMS, particularly in an Indian population.

**Table 3:** 83 patients from various literature documented with DOK-7 mutation causing congenital myasthenia gravis. The table showcases information about the symptoms and the genetic mutation [8,14,16,21,22,23,24]. M= Male, F= Female

Patient no.	Gender	Age of onset	Symptoms of onset	Location of mutation (exon)	Mutation (cDNA)	Mutation (protein)	Reference
1	M	2 years	Frequent falls, respiratory distress, Gower’s sign	7. unknown	c.1124_1127dupTGCC	p.Pro376ProfsX30	[8]
2	F	Birth	Hypotonia	7,4	c.1124_1127dupTGCC, c.346G>A	p.Pro376ProfsX30, p.Val116Met	
3	M	Antenatal	Fetal movement reduction, neonatal respiratory failure	7,1	c.1124_1127dupTGCC, intron 1 inclusiona	p.Pro376ProfsX30, p.Tyr19ValfsX23	
4	M	15 months	Waddling gait	7,4	c.1124_1127dupTGCC, c.437C>T	p.Pro376ProfsX30,p.Pro146Leu	
5	F	Antenatal	Hypotonia, congenital scoliosis, torticollis	7, unknown	c.1124_1127dupTGCC	p.Pro376ProfsX30	
6	M	Birth	Hypotonia	7, unknown	c.1124_1127dupTGCC	p.Pro376ProfsX30	
7	M	15 months	Weakness, frequent falls	7,7	c.1124_1127dupTGCC	p.Pro376ProfsX30	
8	F	15 months	Neck weakness	Intron 6,6	c.773-2A>G	Splice site mutation	
9	F	7 years	Proximal weakness of the lower limbs	7,5	c.1124_1127dupTGCC, c.539G>T	p.Pro376ProfsX30, p.Gly180Val	
10	F	Birth	Hypotonia, stridor, swallowing difficulties	7,4	c.1124_1127dupTGCC, c.470T >G	p.Pro376ProfsX30, p.Leu157Arg	
11	M	<2.5 years	Weakness, ptosis, bulbar symptoms	7,4	c.1124_1127dupTGCC, c.511G>C	p.Pro376ProfsX30, p.Gly171Arg	
12	F	Birth	Stridor, neonatal respiratory distress	7,3	c.1124_1127dupTGCC, c.134C>T	p.Pro376ProfsX30, p.Ser45Leu	
13	F	10 months	Frequent falls	7,4	c.1124_1127dupTGCC, c.514G>A	p.Pro376ProfsX30, p.Gly172Arg	
14	M	13 years	Weakness, Gower’s sign, ptosis	7,7	c.1124_1127dupTGCC, c.1435_1450del16nt	p.Pro376ProfsX30, p.Gly479HisfsX13	
15	M	5 years	Weakness, Gower’s sign	7,7	c.1124_1127dupTGCC, c.1435_1450del16nt	p.Pro376ProfsX30, p.Gly479HisfsX13	
16	F	When started to walk	Motor problems, ptosis	-	1263insC—homozygous	-	[14]
17	F	Infancy	Weakness,Fatigue	-	1124_1127dupTGCC—heterozygous	-	
18	M	Birth	Weakness	-	1263insC—homozygous	-	
19	M	Birth	Acute respiratory failure, stridor	-	1124_1127dupTGCC—heterozygous	-	
20	M	Birth	Hypotonia	-	1124_1127dupTGCC—heterozygous	-	
21	M	6 years	Generalized weakness	4,7	480CA stop—heterozygous	-	
22	M	2-3 year	Frequent falls, waddling gait	Exon 4 Exon 7	396C>G 1124_1127dupTGCC	H132Q P376P fsX30	[16]
23	M	childhood	Problems when doing sports	Exon 4 Exon 7	396C>G 1124_1127dupTGCC	H132Q P376P fsX30	
24	F	Birth	Respiratory distress, hypotonia	Exon 7 Exon 7	1378insC 1508insC	E460P fsX58 P503P fsX15	
25	F	3 year	Frequent falls	Exon 7 Exon 7	1124_1127dupTGCC 1263insC	P376P fsX30 S422L fsX94	
26	F	2 year	Frequent falls	Exon 7 Exon 7	1124_1127dupTGCC 1357_1370del14	P376P fsX30 R452R fsX61	
27	F	2 year	Frequent falls	Intron 2 Exon 7	IVS2-1G>T 1124_1127dupTGCC	*Only mother’s DNA was available P376P fsX30	
28	M	12 year	Abnormal fatigability	Exon 7 Exon 7	1124_1127dupTGCC 1124_1127dupTGCC	P376P fsX30 P376P fsX30	
29	M	Birth	Hypotonia	Exon 5 Exon 7	601C>T 1124_1127dupTGCC	R201X P376P fsX30	
30	M	22 year	Walking Difficulties	Exon 7 Exon 7	1124_1127dupTGCC 1124_1127dupTGCC	P376P fsX30 P376P fsX30	
31	F	25 year	Walking Difficulties	Exon 7 Exon 7	1124_1127dupTGCC 1124_1127dupTGCC	P376P fsX30 P376P fsX30	
32	F	2 year	Walking difficulties	Exon 5 Exon 7	555delC 1296_1311del16	S185S fsX59 R432R fsX17	
33	F	Birth	Hypotonia	Exon 2 Exon 7	98G>A 1124_1127dupTGCC	A33V P376P fsX30	
34	M	1 year	Motor mile stones delayed	Exon 3 Exon 7	134C>T 1124_1127dupTGCC	S45L P376P fsX30	
35	F	Birth	Hypotonia	Exon 3 Exon 7	134C>T 1406C>A	S45L P469H	
36	M	birth	Feeding problems and SOB	-	c.1124_1127dupTGCC c.1339_1342dupCTGG	-	[21]
37	F	1-2 year	difficulty walking	7	c.1124_1127dupTGCC c.1263insC	-	
38	F	birth	Ptosis	7	c.1124_1127dupTGCC c.1263insC	-	
39	M	2 year	difficulty walking	7,7	c.1124_1127dupTGCC c.1124_1127dupTGCC	-	
40	M	3year	difficulty walking	7	c.1124_1127dupTGCC-	-	
41	M	5-6 year	difficulty walking	-	c.593G4C c.1124_1127dupTGCC	-	
42	F	1.5-2 year	difficulty walking	-	c.593G4C c.1124_1127dupTGCC	-	
43	F	birth	floppy infant	7	c.1124_1127dupTGCC-	-	
44	F	birth	floppy infant	7	c.1124_1127dupTGCC-	-	
45	F	3 year	difficulty walking	-	c.473G4A c.1124_1127dupTGCC	-	
46	F	3-4 year	difficulty walking	-	c.1124_1127dupTGCC c.1124_1127dupTGCC	-	
47	F	1 year	difficulty walking	-	c.1124_1127dupTGCC c.1263insC	-	
48	F	birth	floppy infant, feeding problems	-	c.1124_1127dupTGCC c.1124_1127dupTGCC	-	
49	F	1.5 year	difficulty walking	-	c.1143insC c.1143insC	-	
50	F	2-3 year	ptosis and lordosis	-	c.548_551delTCCT c.1124_1127dupTGCC	-	
51	-	Birth	vocal cord palsy/ weakness	-	-	-	[22]
52	-	Birth	vocal cord palsy/ hypotonia	-	-	-	
53	-	Birth	vocal cord palsy	-	-	-	
54	-	Birth	stridor	-	-	-	
55	-	36 months	weakness	-	-	-	
56	-	Birth	feeding difficulties,hypotonia	-	-	-	
57	-	10 months	axial weakness	-	-	-	
58	-	Birth	stridor	-	-	-	
59	-	Birth	vocal cord palsy	-	-	-	
60	-	18 months	weakness	-	-	-	
61	-	Birth	stridor	-	-	-	
62	-	Birth	vocal cord palsy	-	-	-	
63	-	Birth	feeding difficulties/motor delay	-	-	-	
64	-	14 months	proximal weakness	-	-	-	
65	M	12 years	bilateral proximal weakness involving both upper and lower limbs, difficulty in standing up and lifting heavy objects	-	-	NM_173660.4: c.379G > A, p.(Gly127Ser)	[23]
66	F	24 years		-	-		
67	F	19 years		-	-		
16 patients in total, 8 female and 8 male (83)		First day of life to 5 years of age.	Easy fatigability on exertion. Short lived exacerbations lasting from days to weeks. Respiratory embarrassment as a severe complication. They may perceive decreased fetal movements and experience proximal and distal limb weakness. Eyelid ptosis, ocular paresis, facial weakness and bulbar symptoms might also be present. All show characteristic decrement on EMG.	-	596delT 1124_1127dupTGCC	Ile199ThrfsX47 Ala378SerfsX30	[24]
				-	Intron 1 inclusiona 1124_1127dupTGCC	Tyr19ValfsX23 Ala378SerfsX30	
				-	1263delC 1124_1127dupTGCC	Ser422HisfsX34 Ala378SerfsX30	
				-	1124_1127dupTGCC 1124_1127dupTGCC	Ala378SerfsX30 Ala378SerfsX30	
				-	Intron 1 inclusiona 1124_1127dupTGCC	Tyr19ValfsX23 Ala378SerfsX30	
				-	601C T 1124_1127dupTGCC	Arg201X Ala378SerfsX30	
				-	1263insC 1124_1127dupTGCC	Ser422LeufsX97 Ala378SerfsX30	
				-	55-2A C (IVS1-2A C) 1124_1127dupTGCC	Trp19ValfsX70 Ala378SerfsX30	
				-	1001_1011dup 101_652del	Ser338AlafsX122 Ex3-6 skipping	
				-	55_100delb 1124_1127dupTGCC	Ex2 skipping Ala378SerfsX30	
				-	1263insC 1124_1127dupTGCC	Ser422LeufsX97 Ala378SerfsX30	
				-	331 1G T (IVS3 1G T)b 1124_1127dupTGCC	Ex3 skipping Ala378SerfsX30	
				-	1139_1141delinsA 1513T C	Ala380AspfsX76 X505Argext183	
				-	1378insC 1124_1127dupTGCC	Gln460ProfsX59 Ala378SerfsX30	
				-	1263insC 1124_1127dupTGCC	Ser422LeufsX97 Ala378SerfsX30	
				-	1263insC 1124_1127dupTGCC	Ser422LeufsX97 Ala378SerfsX30	

## Conclusions

Based on the above-mentioned findings, CMS can present either in the neonatal period, infancy, or up to two years of age. The clinical picture of CMS can vary greatly. Typically, early-onset subgroups of the DOK-7 mutation have more severe disease manifestations. In DOK-7 CMS, oral salbutamol appears to be well tolerated and enhances muscle function. Salbutamol is preferred over ephedrine because ephedrine has central and cardiac side effects with long-term use.
